# Feasibility and Acceptability of Teleconsultation During COVID-19: A Cross-Sectional Study

**DOI:** 10.7759/cureus.30937

**Published:** 2022-10-31

**Authors:** Manish Raj, Priyanka Rai, Narasimha G V L, Abhishek Onkar, Sumeet Angral, Saurabh Varshney

**Affiliations:** 1 Department of Orthopaedics, All India Institute of Medical Sciences, Deoghar, IND; 2 Department of Obstetrics and Gynaecology, All India Institute of Medical Sciences, Deoghar, IND; 3 Department of Psychiatry, All India Institute of Medical Sciences, Deoghar, IND; 4 Department of Ophthalmology, All India Institute of Medical Sciences, Deoghar, IND; 5 Department of Otolaryngology, All India Institute of Medical Sciences, Deoghar, IND

**Keywords:** covid-19, patient satisfaction, acceptability, feasibility, telemedicine

## Abstract

Background

The coronavirus disease 2019 (COVID-19) pandemic created an aberrant challenge for healthcare delivery systems, forcing public health policies across the globe to be shifted from traditional medical care in hospitals to virtual care in the homes of patients. To tackle this pandemic, telemedicine had taken center stage. This study aims to learn about patient satisfaction, feasibility, and acceptability of the use of telemedicine for clinical encounters during the COVID-19 pandemic.

Methodology

This single-center, cross-sectional, observational study was done on a total of 758 patients who were provided with teleconsultations during the COVID-19 pandemic. We developed a 49-item questionnaire consisting of patients’ quality of consultation and patients’ expectations to evaluate the feasibility, acceptability, and patient satisfaction with their telemedicine consultations.

Results

The majority of survey participants (97.1%) expressed satisfaction with the quality of the consultations provided through telemedicine. A large percentage of participants (96.8%) reported the benefits of teleconsultation in treating their problems. Overall, 93.3% of participants responded positively to the continuation of teleconsultation services after the pandemic.

Conclusions

The study revealed a wide extent of satisfaction among patients. The feasibility and acceptability of telemedicine services have transformed the mode of healthcare delivery systems.

## Introduction

On February 11, 2020, the World Health Organization (WHO) officially declared that the coronavirus outbreak that began in 2019 be known as coronavirus disease 2019 (COVID-19) [1], and the novel coronavirus was named severe acute respiratory syndrome coronavirus 2 (SARS-CoV-2) by the International Committee on Taxonomy of Viruses [2]. The sudden emergence of this unknown virus with no specific treatment guidelines led to prioritizing preventive measures such as containment and isolation of COVID-19 patients at home to prevent the transmission of viruses. Globally, various public health policies implemented quarantine and isolation protocols to stop the transmission of the coronavirus into more communities. Due to this quarantine policy, there was a paradigm shift in medical care from hospitals to the homes of patients. The services of telemedicine were utilized as a first-line tool to elude the transmission of this pandemic [3-6]. Globally, telemedicine has been used in various forms over the last few decades [7-10]. However, the unwillingness to implement telemedicine by medical providers and patients themselves coupled with the absence of a valid legal framework and a lack of financial investment in technological resources for operating telemedicine services in hospitals led to the slow growth of telemedicine [11]. The COVID-19 pandemic provided the ideal platform for promoting and accelerating the implementation of digital technologies, such as telemedicine, to face this emergency.

Consultations for COVID-19 through telemedicine not only provided isolated individuals with timely information but also helped prevent loneliness, stress, and anxiety. This also helped patients to maintain a sense of social belonging that improved their physical symptoms [12]. Hospitalization involves a loss of independence; however, home care involves a familiar environment such as own room, bed, and other home amenities. However, research on the feasibility and acceptability of such services has been minimal in the past [13-16]. The primary objective of this study was to assess the feasibility and acceptability of telemedicine services during COVID-19. The secondary objective was to assess the profile and clinical characteristics of patients who participated in teleconsultation along with outcomes.

## Materials and methods

**Figure 1:**
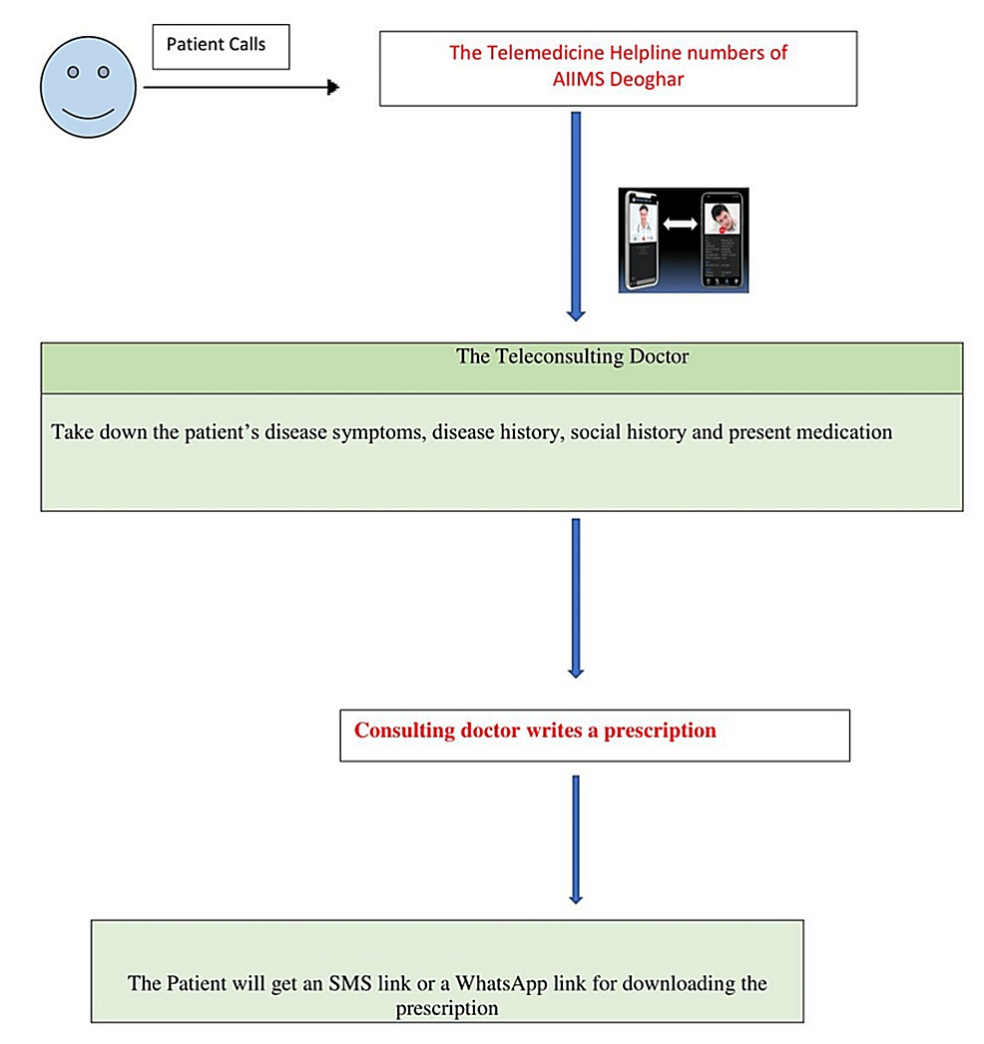
Diagrammatic representation of the telemedicine services of All India Institute of Medical Sciences, Deoghar.

This was a descriptive, cross-sectional, observational study with a questionnaire-based design conducted between August 2021 and August 2022 at a government-run, tertiary care teaching hospital in the largest tribal area of India. To provide easily accessible and uninterrupted medical consultations and care to patients during the lockdown that resulted in the complete closure of outpatient department services throughout the country, the All India Institute of Medical Sciences (AIIMS), Deoghar used COVID-19 teleconsultation services that had already been established by the telemedicine department of the institute. We provided teleconsultation through two telephone numbers dedicated to COVID-19 consultation every day via audio, video, or hybrid modes to patients who registered themselves between 9 am and 5 pm. Treatment protocols were set based on national guidelines and updated regularly. Clinicians from different specialties were posted in COVID-19 teleconsultation services and received regular training on COVID-19 treatment protocols. Regular training programs on updated treatment schedules were conducted. Case discussions occurred regularly to enhance case-based learning. We used two tablets with 4G sims and an internet facility to access popular messaging and video-calling platforms such as WhatsApp to provide teleconsultations. Treatment plans were written electronically, and prescriptions were sent to patients over WhatsApp. During teleconsultations, if clinicians felt there was an immediate need for the hospitalization of a patient, they were directed to the nodal officer of the COVID-19 control center for hospitalization and inpatient care. For proper follow-up, patients were encouraged to call back for teleconsultation continuity or resolve any new query. The different clinical complaints and details of the patients were noted by the clinician during teleconsultations. The confidentiality of patient data was maintained, and telecommunication was end-to-end encrypted according to the strict protocols of the Information Technology Act of India, 2000 and the Personal Protection Data Bill, 2019. We designed an appropriate tool in the form of a questionnaire to evaluate the feasibility, acceptability, and impact of telemedicine services of AIIMS Deoghar. The 49-item questionnaire was developed which included questions related to patients’ sociodemographic details, teleconsultation details, and expectations from telemedicine consultations, as well as the patients’ rating on the quality of the teleconsultation validated by two experts in the field. All patients who had availed of telemedicine services were prospectively requested to enroll in this study after obtaining consent. All data were collected on Google Forms. Feasibility criteria for this study were considered if the satisfaction with the quality of the consultation was greater than 50% among survey participants. Acceptability was defined as more than 50% of the participants responding positively to the continuation of the services. The study was approved by the Institutional Ethics Committee of AIIMS Deoghar (approval number: 2021- 01-IND-01). Figure 1 shows a diagrammatic representation of the telemedicine services of AIIMS Deoghar.

## Results

Sociodemographic details

A total of 758 patients agreed to be contacted after responding to a questionnaire related to the feasibility and acceptability of telemedicine consultations during COVID-19. A large proportion of survey participants (713, 94%) reported good to excellent access to coronavirus services, with local health centers located within a few kilometers. Vaccination services for survey participants were reported to be highly available to 740 (97.6%) participants compared to only 1.1% of participants who reported non-availability of vaccination services at their health centers. Almost two-thirds of survey participants (72.8%) reported they had no prior experience of consulting a doctor through telemedicine. Table 1 presents the sociodemographic variables of the study population.

**Table 1:** Sociodemographic variables of the study population.

Variables	Groups	n (%)
Occupation	Skilled	441 (58.2%)
	Semiskilled	129 (17% )
	Unskilled	188 (24.8% )
Educational status	Primary	85 (11.2% )
	Secondary	153 (20.25%)
	Undergraduate and above	520 (68.6%)
Income	Below poverty line	348 (45.9%)
	Above poverty line	410 (54.1%)
Area of living	Urban	623 (82.2%)
	Rural	135 (17.8%)
Sex	Male	517 (68.2%)
	Female	241 (31.8%)

Feasibility and acceptability of telemedicine services

Overall, 97% of participants were highly satisfied with the communication of the doctor during teleconsultation. Only two (0.3%) patients were not satisfied with the doctor’s communication. The majority of participants (97.7%) in the survey reported being given adequate time during consultation to state their queries, and only two (0.3%) participants were not satisfied with the consulting time given to them. Most participants (97.1%) reported that consulting doctors had completely understood and addressed all of their concerns regarding the disease, vaccination, medication, and investigation during teleconsultation, and only four (0.5%) participants had reported that consulting doctors had not clearly understood all of their concerns. During the survey, 85.5% of patients reported they had followed the advice given by doctors on the COVID-19 helpline. When participants were asked whether teleconsultations treated their problems, the majority of the participants (96.8%) responded positively, showing the feasibility of these teleservices. Only seven (0.9%) participants reported that teleconsultation did not benefit them at all. Regarding the continuity of these teleconsultation services, 93.3% of participants expected these telemedicine services to be continued after the pandemic, showing the acceptability of these teleconsultation services. Table 2 presents the nature of help-seeking and access to care, and Table 3 presents the feasibility and acceptability of telemedicine services.

**Table 2:** Nature of help-seeking and access to care. COVID-19: coronavirus disease 2019

Variables	Groups	n (%)
Index person for whom help was sought	Myself	613 (80.9%)
	Family member	145 (19.1%)
Reason for seeking help	COVID-19	122 (16.1%)
	Non-COVID19	525 (69.3%)
	Vaccine	111 (14.6%)
In case you have called for a COVID-19-positive patient, what was the reason for calling?	COVID-19 positive	53 (7%)
	Symptoms of COVID-19 positive	90 (12%)
	Not a COVID-19-related call	610 (81%)
How far is the nearest healthcare facility to your place?	0–10 km	684 (90.2%)
	10–50 km	58 (7.7%)
	>50 km	16 (2.1%)
Are you vaccinated for COVID-19?	First dose	267 (35.2%)
	Both dose	273 (36 %)
	No	218 (28.8%)
What is the type of consultation for seeking help (to be explained to the caller)?	Primary consultation (index opinion)	226 (29.8%)
	Secondary consultation (second opinion)	531 (70.1%)
	Referral from others	00 (0%)
How did you come to know about the telemedicine helpline?	Social media (Twitter, WhatsApp, Facebook, Instagram)	383 (50.5%)
	Print media (newspaper)	336 (44.3%)
	Audio-visual media (television, radio)	6 (0.8%)
	Relatives, friends	33 (4.4%)
	Patients	00 (0%)

**Table 3:** Feasibility and acceptability of telemedicine services.

Variables	Groups	n (%)
Have you ever consulted a doctor over telemedicine in the past?	Yes	206 (27.2%)
	No	552 (72.8%)
If yes, compared to the earlier service how satisfied are you with this service?	Better than the previous one	52 (6.9%)
	Same as the previous one	118 (15.7%)
	Worse than the previous one	9 (1.2%)
	Not applicable	571 (76.1%)
Did you follow the advice given by the AIIMS helpline?	Yes	651 (85.9%)
	No	78 (10.3%)
	Partially	28 (3.7%)
Are you worried about privacy and confidentiality?	Yes	67 (8.8%)
	No	544 (71.8%)
	Not sure	147 (19.4%)
In which language do you expect your doctor should communicate with you?	Hindi	727 (95.9%)
	English	13 (1.7%)
	Local language	7 (0.9%)
	Mixed	11 (1.5%)
Do you want this consultant to be free or paid?	Free	667 (88%)
	Paid	91 (12%)
If the service is a paid consultation, how much would you like to pay?	0–50 Rs	90 (11.9%)
	51–100 Rs	15 (2%)
	>101 Rs	4 (0.5%)
	Not applicable	648 (85.6%)
What medium of consultation do you prefer?	Video consultation	515 (67.9%)
	Telephonic	243 (32.1%)
If we provide video consultation, will you be able to access the service?	Yes	714 (94.2%)
	No	44 (5.8%)
If yes, please provide the medium (here we are providing common mediums used)	Facebook	101 (13.4%)
	WhatsApp	642 (84.9%)
	Instagram	4 (0.5%)
	Google Meet	00 (0%)
	Zoom	00 (0 %)
	Not applicable	8 (1.1%)
	No use of anything	00 (0%)
Which kind of consultation do you prefer?	Telemedicine	200 (26.4%)
	Face to face	376 (49.6%)
	Combination	182 (24%)
Do you expect the prescription to be delivered to you during teleconsultation?	Yes	747 (98.7%)
	No	10 (1.3%)
If yes, what route do you prefer?	Social media like WhatsApp	742 (98.1%)
	Postal service	12 (1.6%)
	No	00 (0%)
	Do not want prescription	00 (0%)
Do you expect medicine to be given to you along with the prescription?	No, I will buy my own	290 (38.3%)
	Yes, at a subsidized rate	379 (50.1%)
	Yes, free of cost	85 (11.2%)
	According to the patient’s economic status	-
	It should be according to government policy	00 (0%)
	Do not want prescription	00 (0%)
Do you think investigations should be ordered over telemedicine and reviewed on follow-up?	Yes	749 (98.9%)
	No	8 (1.1%)
Do you want telemedicine services to be continued after the pandemic?	Yes	707 (93.3%)
	No	27 (3.6%)
	Not sure	24 (3.2%)
What kind of service do you expect in telemedicine?	Primary care	101 (13.3%)
	Specialist	647 (85.5%)
	Referral service	3 (0.4%)
	Not sure	5 (0.7%)
What should be the timing of the availability of telemedicine services?	Round the clock	299 (39.5%)
	Working	415 (54.8%)
	Non-working	35 (4.6%)
	Not sure	8 (1.1%)

## Discussion

Telemedicine simply means patients consulting with a doctor for treatment through a virtual mode [17]. Before COVID-19, there was a slow growth of telemedicine due to limiting factors such as a lack of physical examination, non-availability of diagnostic testing or imaging, and various technical support issues [18,19]. Due to the high transmission and infection rates of the coronavirus, telemedicine took center stage in the management of patient healthcare during the COVID-19 pandemic [20]. In the last week of March 2020, a complete lockdown was announced in India for a few weeks in an attempt to break the chain of infection and contain the COVID-19 pandemic. The majority of non-emergency healthcare, such as outpatient clinics and hospitals, were converted to dedicated COVID-19 hospitals. Due to a large number of COVID-19-positive patients and a lack of available beds at COVID-19-dedicated hospitals, the Government of India encouraged home isolation of COVID-19-affected patients. The policy of home isolation was based on the finding that the majority of COVID-19 patients were either asymptomatic or had very mild symptoms. These cases usually do not require hospitalization in COVID-19-dedicated hospitals and can be managed at home under proper medical guidance and monitoring. This shifted the policy of COVID-19 management from COVID-19-dedicated hospitals to virtual management through different modes of teleconsultation.

A 24-hour service was provided for doctors and patients to communicate via a digital tool such as video calling. Even for patients who recovered from a COVID-19 infection, there was a need for medical care without exposure to other patients, which generally occurs during transportation or hospital consultations. Only a few prior studies have researched the perceptions of patients after telemedicine consultations. There was also a need to study the usefulness, acceptance, and satisfaction of telemedicine services by patients. In the past, few studies have assessed the quality of telehealth services and related patient satisfaction [21-24]. The concept of satisfaction is debatable. Some authors relate satisfaction to individual perceptions of the outcomes of care [25]. Abdel et al. conducted a cross-sectional survey study on 425 patients measuring their satisfaction with telemedicine used in Saudi Arabia during the COVID-19 pandemic and found that patients were satisfied with and positive toward telemedicine programs in Saudi Arabia [13]. Alromaihi et al. evaluated patient satisfaction and utilization of telemedicine services during COVID-19 on 901 participants and found a high level of satisfaction among patients. Overall, 56.6% of participants preferred to continue using telemedicine after the COVID-19 pandemic [14]. Baudier et al. conducted a questionnaire-based study to investigate the acceptance of teleconsultation services by patients during the COVID-19 pandemic. The study had over 386 respondents across several countries in Europe and Asia. The study highlighted the larger impact of performance expectancy, the negative influence of perceived risk, and the positive influence of contamination avoidance with the adoption of teleconsultation solutions [15]. Ramaswamy et al. conducted a retrospective, observational cohort study on 38,609 patients to determine if patient satisfaction differed between video consultations and traditional in-person clinic visits. The study found that patient satisfaction with video visits was high and not a barrier to a paradigm shift away from traditional in-person clinic visits [24]. Hong et al. investigated the population-level volume of internet searches for telehealth during the COVID-19 pandemic in the United States. They found that as the number of COVID-19 cases increased, so did the population’s interest in telehealth, with a strong correlation between population interest and COVID-19 cases reported (r = 0.948, p < 0.001) [22]. D’Souza et al. conducted a study in a tertiary health center in South India on telemedicine services during the COVID-19 pandemic and reported that a majority (95.39%) of patients were satisfied with the consultation rendered through telemedicine during the COVID-19 pandemic [16]. In our study, the majority of the participants (96.8%) expressed satisfaction with the teleconsultation provided to them during the COVID-19 pandemic and wished to continue them in the near future, illustrating the feasibility and sustainability of teleconsultation services.

A major limitation of telemedicine includes privacy and confidentiality issues along with technological glitches and medico-legal issues. One of the major limitations of our study was its single-center design with the absence of a control group (in-person consultation due to the closure of hospitals during the COVID-19 pandemic) while assessing the patient’s satisfaction and willingness to use telemedicine services.

## Conclusions

Understanding a patient’s attitude, perception, and satisfaction with virtual consultation is the most important factor in the success and development of telemedicine healthcare. The results of our study suggest a high satisfaction level among users of telemedicine, with the majority wanting to continue in the near future, showing the feasibility and acceptability of telemedicine services. However, a multicentric study comparing telemedicine with other moded of healthcare delivery systems is needed in the future.
